# Reproducibility of liver R2* quantification for liver iron quantification from cardiac R2* acquisitions

**DOI:** 10.1007/s00261-021-03099-4

**Published:** 2021-05-12

**Authors:** M. R. Muehler, K. Vigen, D. Hernando, A. Zhu, T. J. Colgan, S. B. Reeder

**Affiliations:** 1 Department of Radiology, Wisconsin Institutes of Medical Research, University of Wisconsin, Room 2478, 1111 Highland Avenue, Madison, WI 53705 USA; 2 Department of Radiology and Neuroradiology, University Greifswald, Greifswald, Germany; 3 Department of Biomedical Engineering, University of Wisconsin, Madison, WI USA; 4 Department of Medical Physics, University of Wisconsin, Madison, WI USA; 5 Department of Electrical and Computer Engineering, University of Wisconsin, Madison, WI USA; 6 Department of Medicine, University of Wisconsin, Madison, WI USA; 7 Department of Emergency Medicine, University of Wisconsin, Madison, WI USA

**Keywords:** R2* mapping, Liver iron, 2D cardic, 3D liver

## Abstract

**Objectives:**

To evaluate the reproducibility of liver R2* measurements between a 2D cardiac ECG-gated and a 3D breath-hold liver CSE-MRI acquisition for liver iron quantification.

**Methods:**

A total of 54 1.5 T MRI exams from 51 subjects (18 women, 36 men, age 35.2 ± 21.8) were included. These included two sub-studies with 23 clinical MRI exams from 19 patients identified retrospectively, 24 participants with known or suspected iron overload, and 7 healthy volunteers acquired prospectively. The 2D cardiac and the 3D liver R2* maps were acquired in the same exam. Either acquisitions were reconstructed using a complex R2* algorithm that accounts for the presence of fat and residual phase errors due to eddy currents. Data were analyzed using colocalized ROIs in the liver.

**Results:**

Linear regression analysis demonstrated high Pearson’s correlation and Lin’s concordance coefficient for the overall study and both sub-studies. Bland–Altman analysis also showed good agreement, except for a slight increase of the mean R2* value above ~ 400 s^−1^. The Kolmogorow–Smirnow test revealed a non-normal distribution for (R2* 3D–R2* 2D) values from 0 to 600 s^−1^ in contrast to the 0–200 s^−1^ and 0–400 s^−1^ subpopulations. Linear regression analysis showed no relevant differences other than the intercept, likely due to only 7 measurements above 400 s^−1^.

**Conclusions:**

The results demonstrate that R2*-measurements in the liver are feasible using 2D cardiac R2* maps compared to 3D liver R2* maps as the reference. Liver R2* may be underestimated for R2* > 400 s^−1^ using the 2D cardiac R2* mapping method.

## Introduction

Iron is a major component of essential proteins like hemoglobin and myoglobin. Under normal circumstances, the body iron is maintained in a narrow homeostatic range based primarily on the rate of absorption [1, 2]. Normal elimination occurs through sloughing of intestinal lining cells and skin cells, and by menstruation [2]. Otherwise, humans are incapable of modifying iron excretion without medical intervention, which can lead to iron overload [2, 3].

The most common cause for primary iron overload is hereditary hemochromatosis (HH), occurring in the Caucasian population with a prevalence between 0.2 and 0.45%. Iron overload from HH results from disruption in the hepcidin pathway, with subsequent excess absorption of iron from the gut [1, 4]. Increased intestinal uptake can lead to systemic iron overload after saturation of endogenous ferritin and transferrin [1], leading to free iron that is oxidative and can cause cellular injury [1]. Causes of secondary iron overload, e.g., hemosiderosis, include chronic hemolysis, repeated blood transfusions, myelodysplastic syndrome, among other causes. Comorbidities of iron overload include liver and pancreatic injury, iron overload cardiomyopathy, arthropathy, and growth failure [5–8].

Serum ferritin is most commonly used to assess body iron content closely correlating with total liver iron content [9]. However, ferritin is also an acute-phase reactant, rising with inflammation and declining with ascorbate deficiency confounding and limiting its clinical utility to detect iron overload and for treatment monitoring [5, 10–13]. Transferrin saturation is another serologic marker but in cases exceeding 85% iron saturation assessment of chelation therapy efficiency fails [13].

Historically, non-targeted liver biopsy has been the reference standard for liver iron concentration (LIC) determination [2, 14]. In addition to being an invasive procedure, it is also a destructive test, when relying on atomic absorption spectroscopy, obviating histological examination [15]. Alternatively, a semiquantitative scale-based assessment by staining with Perl’s Prussian Blue is available but is less precise [1].

Magnetic resonance imaging (MRI) is exquisitely sensitive to the presence of iron. Both spin-echo (SE) R2 and gradient echo (GRE)-based R2* relaxation rates increase in the presence of ferritin and hemosiderin [1]. R2 has a monotonic, curvilinear relationship with LIC, and is an established approach to quantify liver iron [16]. SE-based R2 mapping methods require lengthy scan times (10–20 min) and do not provide complete coverage of the liver. R2* has as linear, direct relationship with LIC [1]. Breath-hold GRE-based R2* mapping methods can cover the entire liver in a much shorter acquisition time.

Liver R2* maps obtained using multi-echo spoiled gradient echo (SGRE) chemical shift encoded MRI (CSE-MRI) acquisitions have been proven to be reproducible and more precise than liver biopsy for the quantification of LIC [13–15, 17–20]. These methods are emerging as a rapid, reliable, and reproducible non-invasive method for quantification of LIC and chelation therapy monitoring.

Beyond the liver, other organs including the heart and pancreas can be affected by the presence of iron overload. Severe cardiomyopathy accounts for 71% of deaths in patients with thalassemia major [8, 21]. Quantification of iron content in the pancreas and heart contributes to a complete clinical picture and for therapy planning, and R2* measurements in the heart are important in the management in the patients at risk for iron overload cardiomyopathy [22–24]. R2* mapping in the heart can be performed using dedicated multi-echo 2D cardiac-gated SGRE acquisitions. We note that 2D R2* maps of the heart nearly always include the liver in the field of view (FOV). Consequently, measurement of R2* in the liver is also possible with cardiac-gated 2D acquisitions used to map R2* in the heart.

Therefore, the purpose of this study was to evaluate the in vivo reproducibility of R2* measurements in the liver obtained used ECG-gated 2D cardiac CSE-MRI, and a breath-hold 3D liver CSE-MRI exam with varying acquisition parameters.

## Methods

The reproducibility of R2* measurements in the liver was assessed using two groups of patients. The first group included those patients identified retrospectively who were imaged for clinical purposes to evaluate for known or suspected liver and/or cardiac iron overload. The second group was part of a prospective study performed in patients with known or suspected liver iron overload for the purposes of R2* quantification in the liver [25], in which cardiac R2* mapping was also performed (unpublished). Liver data from the second group that are presented below represent a reanalysis of R2* maps previously acquired and no data from any previous study are replicated here. Both studies were Health Information Privacy and Portability Act (HIPPA) compliant and approved by the local institutional review board (IRB).

### Retrospective clinical study

In the retrospective clinical study, patients who underwent a clinically indicated MRI study for known or suspected iron overload with 2D cardiac and 3D liver CSE-MRI were included. Medical record and PACS review were used to identify cardiac MRI exams with the associated keywords T2*, R2*, iron quantification, IDEAL from 2007 to 2018.

### Prospective research study

Participants over 10 years of age with known or suspected iron overload and with no contraindication for MRI were recruited from the local hematology clinic. In addition, healthy volunteers were recruited from an IRB-approved database of healthy volunteers. Informed written consent was obtained from all subjects recruited for the prospective sub-study.

All subjects were evaluated using both a 3D liver and 2D cardiac CSE-MRI for quantifying R2* of the liver.

### MR acquisition and image reconstruction

All clinical exams included in the retrospective study were performed on clinical 1.5 T MRI systems (Optima MR450w and Signa HDxt, GE Healthcare, Waukesha, WI, USA) using an 8-channel or a 32-channel phased array torso coil. The prospective research study was performed on a clinical 1.5 T MRI system (HDxt, GE Healthcare, Waukesha, WI, USA) using an 8-channel phased array torso coil.

In all studies, a breath-held multi-echo 3D CSE-MRI acquisition with 6–8 echoes covering the entire liver was performed. In addition, a 2D cardiac-gated multi-echo 3D CSE-MRI acquisition with 6–8 echoes was obtained. The field of view (FOV), number and thickness of slices and thus also phase coverage, acquisition matrix, flip angle, and first echo time (TE1) were adapted to the subjects with respect to patient size, heart rate, and hardware constraints (Table 1). An autocalibrated parallel imaging method was used to accelerate the 3D liver MRI acquisition (*R* = 4, outer lines of k-space), as previously described [25, 26]. No parallel imaging acceleration was used for the 2D cardiac MRI R2* mapping acquisition.

**Table 1:** Sequence parameters for the 2D cardiac and 3D liver CSE-MRI of the two studies

Sequence	Retrospective clinical study		Prospective research study	
	3D liver	2D cardiac	3D liver	2D cardiac
Orientation	Axial	Oblique	Axial	Oblique
Slice thickness [mm]	6–8	6–8	8	8
Number of slices	11–72	3–15	32	10
FOV [mm]	350–420 × 140–400	300–420 × 210–360	400 × 360	350–360 × 288–350
Acquired matrix	160–256 × 144–160	192 × 160–192	256 × 160	192–224 × 160–192
Flip angle [°]	5–10	20	5	12–20
TR [ms]	10.1–17.1	18.8–47.5	14.1	15.4–20
Number of echoes	6–8	6–8	6	8
TE1 [ms]	0.8–1.3	1.6–2.2	1.2	1.9–2.1
Echo spacing [ms]	1.4–2.0	1.9–2.6	2.1	1.7–2.2
Parallel imaging acceleration	Yes^a^	No	Yes^a^	No
Acquisition time [min:sec]*	0:15–0:28	0:51–5:28	0:19	2:10–5:06

^a^See MR acquisition and image reconstruction of the Methods section for details

*Note that the acquisition time was obtained from the DICOM header and includes the time for breath-hold commands and breaks between multiple acquisitions

The 2D cardiac CSE-MRI acquisition was acquired in a double-oblique left ventricular short-axis plane that used a FOV that covered the entire heart and also included the liver. Both sequences were acquired in the same exam without repositioning the subjects or phased array coil. No parallel imaging acceleration was used for the cardiac acquisitions.

R2* maps were reconstructed for each of the CSE-MRI acquisitions either 2D or 3D using a previously developed complex R2* mapping algorithm that accounts for the presence of fat and residual phase errors due to eddy currents [27, 28]. The use of complex data obviates the need to correct for bias related to noise floor effects [29].

### Data analysis

**Fig. 1:**
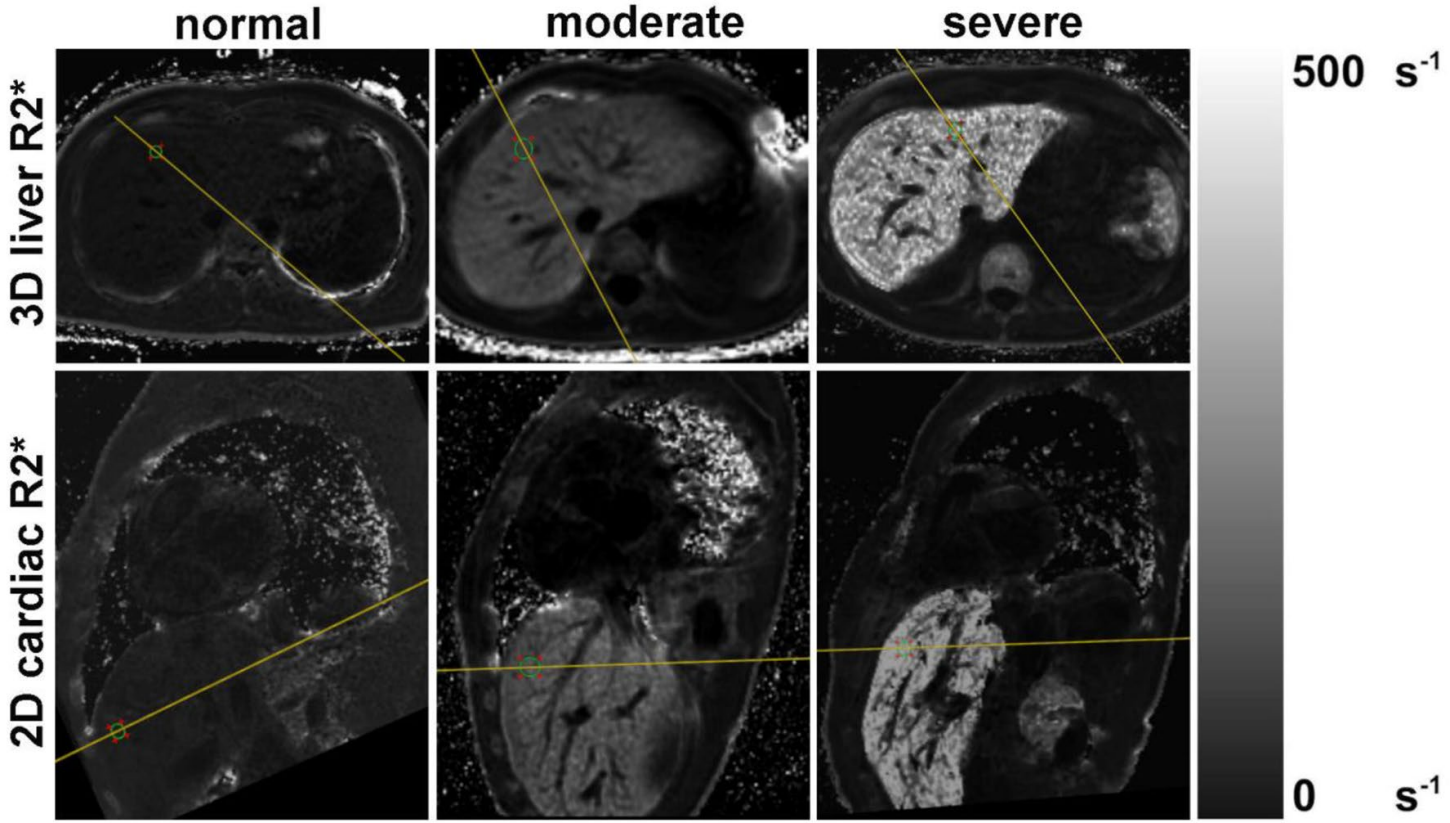
Representative examples of R2* maps in the liver using 3D (top row) and 2D (bottom row) R2* maps from three representative patients, with normal (≈25 s^−1^), moderate (≈150 s^−1^), and severe (≈450 s^−1^) liver iron

Relatively small-sized oval ROIs (~ 0.5–1cm^2^) were placed to achieve close colocalization in the liver on the 2D cardiac and 3D liver R2* maps and to keep their sizes approximately equal in the whole study. ROIs were placed as much as possible in the same overlaying region of the liver in 2D and 3D avoiding large vessels, bile ducts or focal liver lesions, and were drawn using reference lines tool in a side by side hanging in the Image Viewer software, by an experienced radiologist (M.M.) with 20 years of experience in MRI interpretation (Fig. 1).

The imaging studies of the retrospective clinical study were available in PACS (McKesson Radiology Station 12.3, McKesson Medical Imaging Company, Richmond, BC, Canada). ROIs in the 2D cardiac and 3D liver R2* maps were colocalized in the liver using Scroll to Point tool in PACS.

The imaging studies of the prospective research study were available as anonymized, exported DICOM series. ROIs were measured with the RadiAnt DICOM Viewer (version 4.6.9.18463, Medixant, Poznan, Poland, https://www.radiantviewer.com).

### Statistical analysis

Linear regression analysis of the R2* values was performed separately on the retrospective clinical and prospective research studies, as well as on the combined results to assess the correlation between the 2D cardiac and 3D liver R2* measurements. Pearson’s correlation coefficient (r), Lin’s concordance correlation coefficient (ρ), slope and intercept were calculated with their 95% confidence interval (CI). Bland–Altman analysis was used to assess agreement.

The (R2* 3D-R2* 2D) values of three subgroups defined by the mean (R2* 3D, R2* 2D) values from 0 to 200 s^−1^, 0 to 400 s^−1^, and 0 to 600 s^−1^ were assessed for normal distribution using Kolmogorow–Smirnow test. Subgroups were defined empirically with respect to the maximum mean (R2* 3D, R2* 2D) of nearly 600 s^−1^ and an increasing uncertainty of R2* correlation and agreement for values above 400 s^−1^, as seen in Figs. 3 and 4.

Linear regression analysis was also performed for these subgroups as described above.

## Results

**Fig. 2:**
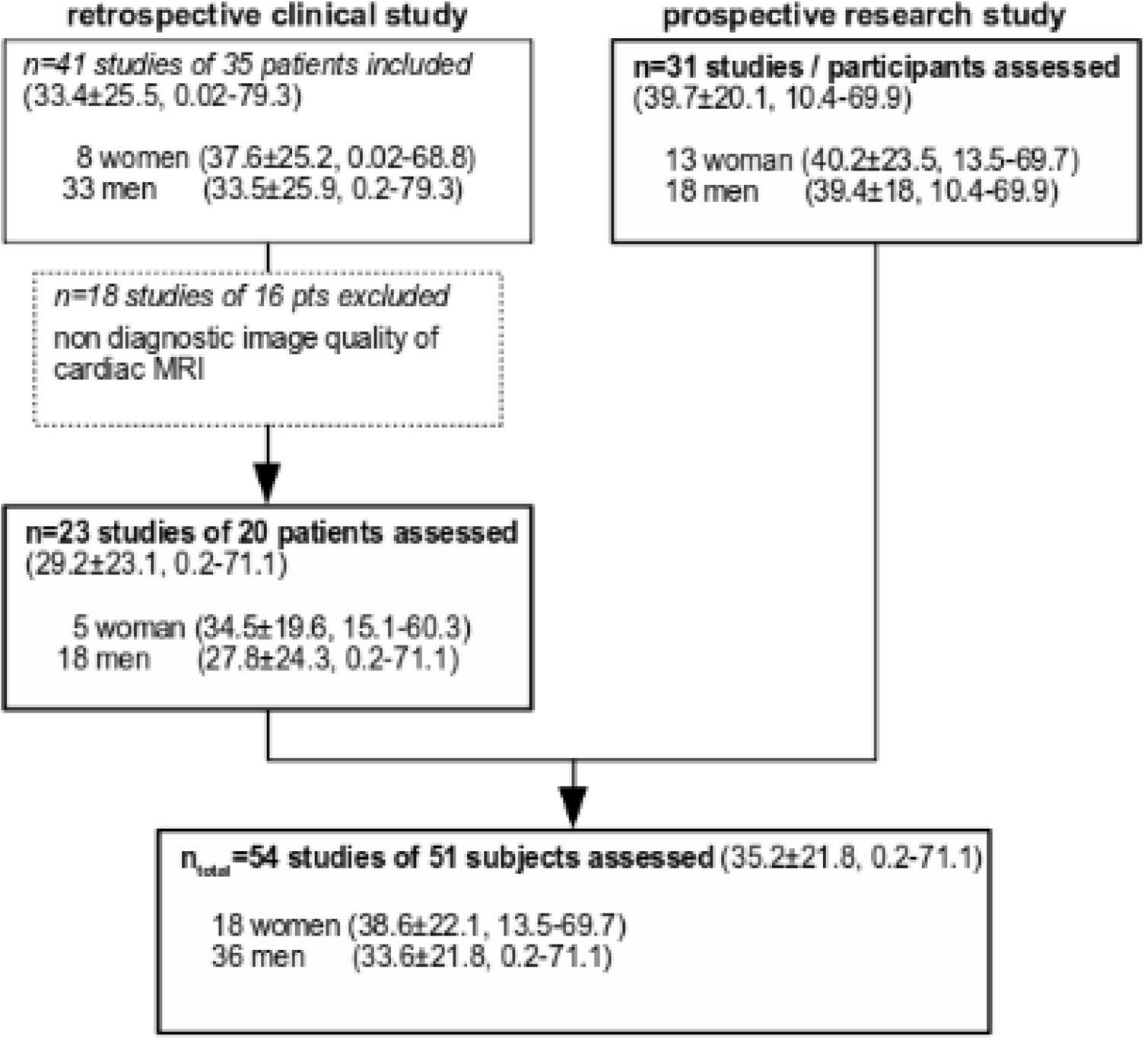
Flowchart depicting inclusion and exclusion of subjects in the study for the retrospective and prospective studies (statement in parentheses is the study data’s mean age ± standard deviation, min–max age)

In total, 51 subjects with a total of 54 MRI exams (18 women, 36 men, age 35.2 ± 21.8) were included in this study, using data combined from the retrospective clinical study and the prospective research study (see Fig. 2 for details).

### Retrospective clinical studies

Following medical records and PACS review, 35 patients with 41 MRI exams (8 women, 33 men, age 34.3 ± 25.5) studies were identified. 18 studies from 16 patients were excluded from the evaluation due to poor diagnostic image quality of the cardiac MRI, technical errors, or lack of patient compliance.

Subsequently, 23 MRI studies from 19 patients performed between August 2010 and October 2018 were assessed in this study (5 women, 18 men, age 29.2±23.1). Three patients (1 woman, 2 men) underwent a single follow-up study between 6 months and 8 years later.

Clinical indications for assessment of cardiac iron overload included hemochromatosis (*n* = 8), leukemia (*n* = 3), thalassemia (*n* = 1), sickle cell anemia (*n* = 2), Blackfan-Diamond anemia (*n* = 2), anaplastic anemia (*n* = 1), gastroschisis (*n* = 1) with elevated ferritin, and ductus arteriosus (PDA) repair (*n* = 1).

### Prospective research study

The prospective research study included 24 participants and 7 volunteers, for a total of 31 subjects (13 female, 18 male, age 39.7 ± 20.1), performed between January 2012 and July 2014, were assessed in this study. No follow-up studies were done in this evaluation study.

Indications for the 24 participants in the prospective research study included hereditary hemochromatosis (*n* = 7), transfusional hemosiderosis in myelodysplastic syndrome (*n* = 3), lymphoma (*n* = 2), acute myeloid leukemia (*n* = 3), chronic lymphatic leukemia (*n* = 1), acute lymphatic leukemia (*n* = 2), sickle cell disease (*n* = 1), dyserythropoietic anemia (*n* = 1), iron deficiency anemia (*n* = 1), aplastic anemia (*n* = 2), and Black-Diamond anemia (*n* = 1).

### Statistical analysis

**Fig. 3:**
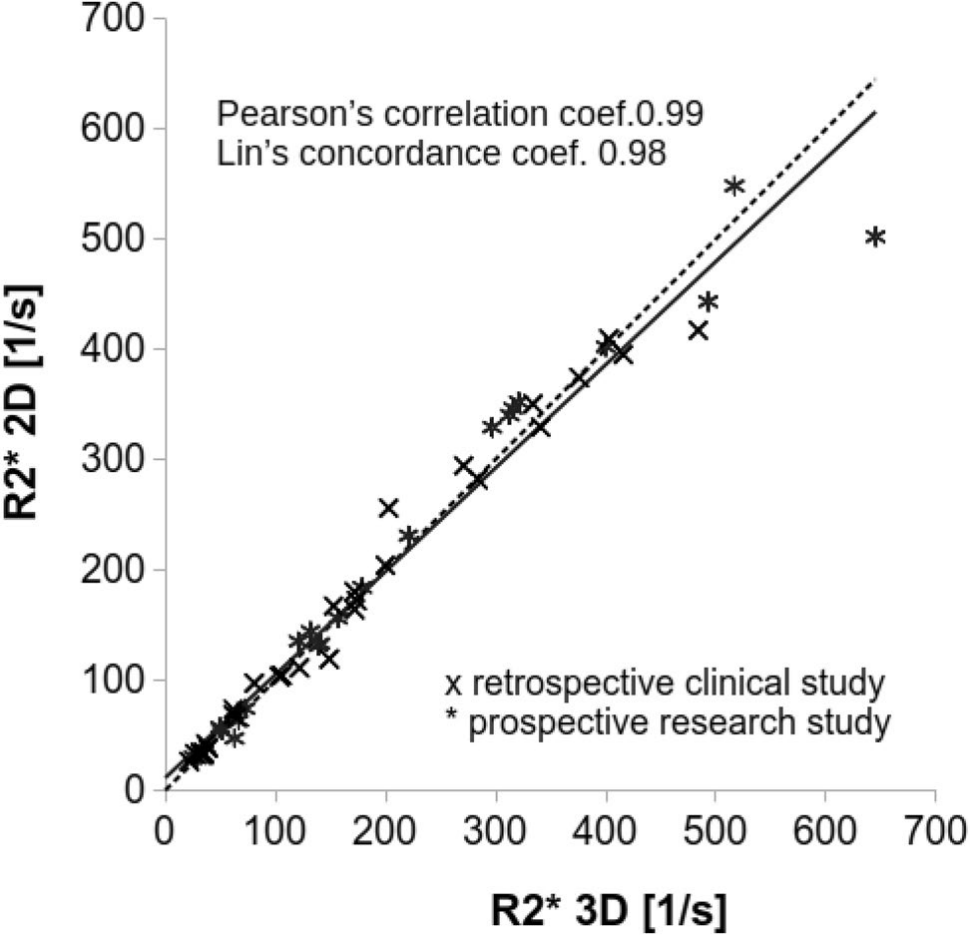
Very strong correlation between R2* measurements made in the liver using 2D CSE-MRI and 3D CSE-MRI was observed. Further, excellent agreement between these methods was observed for R2* values up to 400 s^−1^ (severe iron overload). Results of the linear regression analysis for both the retrospective clinical and prospective research studies together are shown in this diagram. The values closely are located near to y = x (dashed line) indicating low variability of R2* measurements

Linear regression analysis of the R2* values measured using 2D cardiac and 3D liver CSE-MRI demonstrates strong Pearson’s correlation coefficient (*r* = 0.98) and strong Lin’s concordance correlation (ρ = 0.98) for the retrospective clinical study, for the prospective research study (*r* = 0.99 and ρ = 0.99), and for both studies combined (*r* = 0.99 and ρ = 0.98) (Table 2). The linear regression analysis all study groups together is shown in Fig. 3.

**Table 2:** Correlation results for the retrospective study, prospective study, and combined data

	Retrospective clinical study	Prospective research study	Total
Number of acquisitions	23	31	54
Linear regression			
Pearson’s correlation coefficient (95% CI)	0.98 (0.95–0.99)	0.99 (0.98–1.00)	0.99 (0.97–0.99)
Lin’s concordance coefficient (95% CI)	0.98 (0.95–0.99)	0.99 (0.98–1.00)	0.98 (0.97–0.99)
Slope (95% CI)	0.92 (0.84–1.01)	0.96 (0.91–1.01)	0.94 (0.89–0.98)
Intercept (95% CI)	15.9 (− 7.1–38.9)	7.5 (− 3–17.9)	11.3 (0.5–22.2)
Bland–Altman			
Mean of differences (95% CI of mean differences) lower and upper limit of agreement	0.9 (− 13.8–15.6) − 69.6–71.4	− 0.4 (− 7–6.3) − 37.2–36.5	0.2 (− 7.1–7.4) − 53.0–53.4

Shown are correlation coefficients, Lin’s correlation coefficient for all study groups, the slope and interception as well as the mean of differences and the limit of agreement from the Bland–Altman analysis with their 95% confidence intervals (CI)

**Fig. 4:**
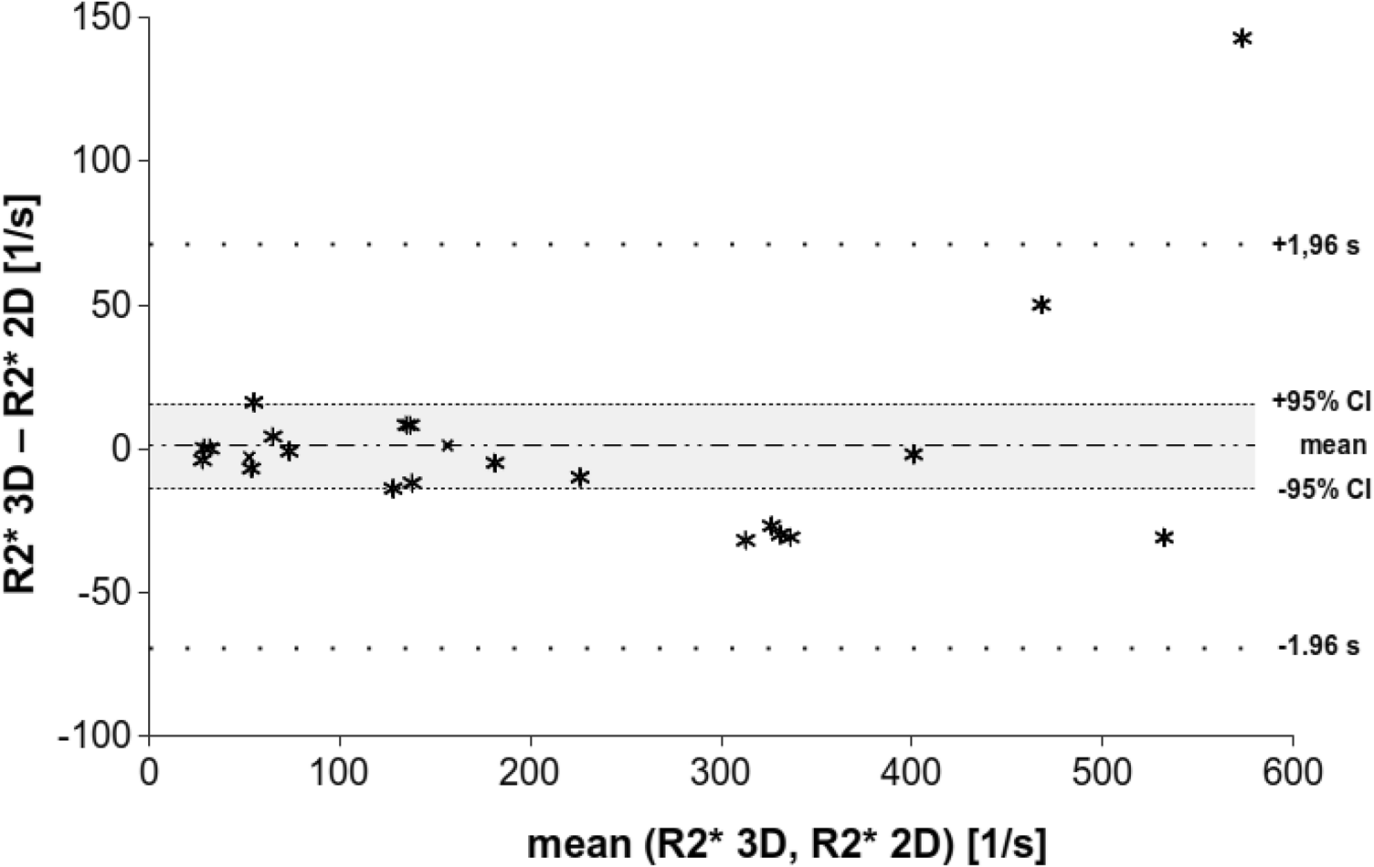
Bland–Altman analysis for the retrospective clinical study (*n* = 23) shows strong agreement between the two R2* mapping methods, particularly at lower levels of iron overload. The mean difference is 0.9 and its 95% confidence interval is ± 14.7 (− 13.8–15.6); the limits of agreement are given (± 70.5, − 69.4–71.4)

**Fig. 5:**
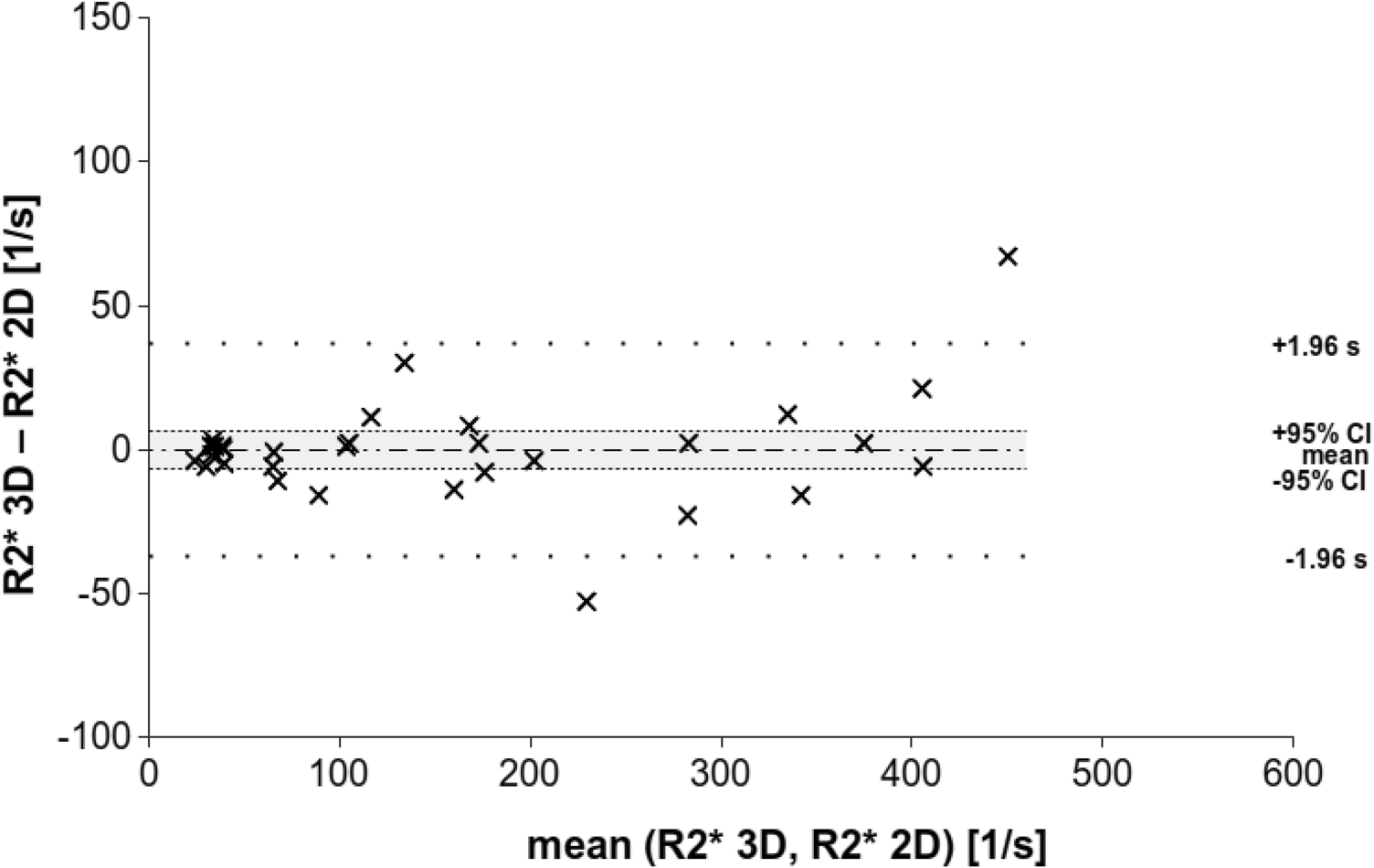
Bland–Altman analysis for the prospective research study (*n* = 31) shows strong agreement between the two R2* mapping methods, particularly at lower levels of iron overload. The mean difference is 0.4, its 95% confidence interval by ± 6.6 (− 7–6.2), and the limits of agreement by ± 36.8 (− 37.2–36.5)

**Fig. 6:**
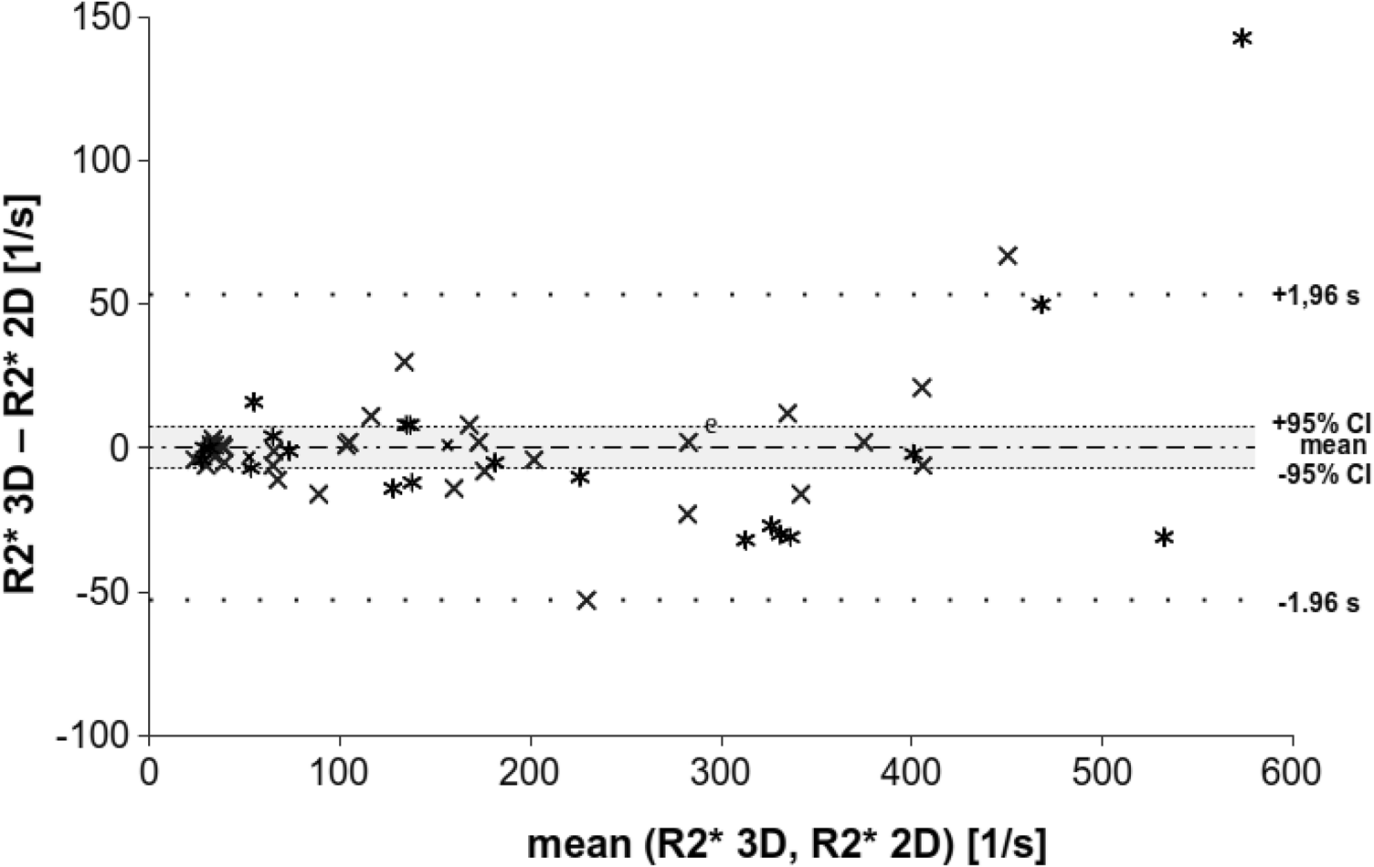
Bland–Altman for both studies together the retrospective clinical and prospective research studies (*n* = 54). The mean difference is 0.2, its 95% confidence interval by ± 7.2 (− 7–7.4), and the limits of agreement by ± 53.2 (− 53.0–53.4) (“x” represents the data from retrospective clinical and “*” represents the prospective research study)

Bland–Altman analysis of the retrospective clinical study shows a mean bias and 95% confidence interval of 1.30 s^−1^ and ± 14.59 s^−1^, for the prospective research study − 0.35 s^−1^ and ± 6.61 s^−1^, and for both studies combined 0.35 s^−1^ and ± 7.2 s^−1^ (Figs. 4, 5, and 6). In all of the groups, the line of equality lies within the mean’s 95% confidence interval indicating no significant bias. In the retrospective study, there is 1 outlying data point out of 23, while in the prospective research there were 2 outlying data points out of 31, for a total of 3 outlying data points out of 54 that exceeded the upper or lower limit of agreement. Thus, about 6% of the data points lay outside of the 95% percentile in the prospective research study and in both studies together.

The Bland–Altman analysis of the combined retrospective clinical and prospective research studies shows that for mean (R2* 3D, R2* 2D) values above about 400 s^−1^ the difference of the R2* values becomes more positive (Fig. 6). This trend indicates that at higher R2* values the 2D acquisition underestimates R2* when compared with the 3D acquisition. The Kolmogorow–Smirnow test for the subgroups of mean (R2* 3D, R2* 2D) values from 0 to 200 s^−1^ and 0 to 400 s^−1^ shows a *p* value of 0.333 and 0.214 (*p* > 0.05), respectively, indicating normally distributed (R2* 3D-R2* 2D) values (Table 3). In contrast, the Kolmogorow–Smirnow test for the subgroup from 0 to 600 s^−1^ shows a *p* value of 0.003 indicating the data differ significantly from the normal distribution. In addition, the Pearson’s correlation coefficient and the Lin’s concordance coefficient were calculated with a value of 0.99, and similar 95% confidence intervals for all the subgroups. (Table 3). Slope and intercept were calculated between 0.94–1.05 and 2.69–11.34, respectively, with some overlap of the 95% confidence intervals (Table 2). The intercept of the subgroup from 0 to 600 s^−1^ was 11.34, higher than for the other groups and in agreement with the Bland–Altman analysis.

**Table 3:** Correlation results over various ranges of iron overload

Mean (R2* 3D, R2* 2D)	(R2^*^ 3D-R2* 2D) values		
	0–200 s^−1^	0–400 s^−1^	0–600 s^−1^
Number of acquisitions	35	47	54
*p* value of Kolmogorow–Smirnow test*	0.333	0.214	0.003
Pearson’s correlation coefficient (95% CI)	0.99 (0.97–0.99)	0.99 (0.99–1.00)	0.99 (0.97–0.99)
Lin’s concordance coefficient (95% CI)	0.99 (0.97–0.99)	0.99 (0.98–0.99)	0.98 (0.97–0.99)
Slope (95% CI)	0.98 (0.92–1.03)	1.05 (1.01–1.09)	0.94 (0.89–0.98)
Intercept (95% CI)	2.69 (− 3.17–8.55)	− 1.86 (− 8.28–4.56)	11.34 (0.50–22.17)

Very strong correlation is noted across all levels of iron overload, however, for R2* values less than 400 s^−1^, excellent agreement with slope and intercept close to 1.0 and 0, respectively, demonstrate excellent reproducibility between 2 and 3D R2* mapping methods. Results of the analysis of the Kolmogorow–Smirnow test of normality, the Pearson’s correlation coefficient, Lin’ concordance coefficient and the slope and interception of the linear regression for three subgroups with R2* measurements are at 0–200 s^−1^, 0–400 s^−1^, and 0–600 s^−1^, respectively. R2* measurements of both the retrospective clinical and the prospective research studies are included in this analysis

*CI* confidence interval

*A *p* value less than 0.05 indicates the data differ significantly from the normal distribution

## Discussion

In this study, we evaluated the in vivo reproducibility of R2* measurements in the liver between two different R2* mapping approaches, either an ECG-gated 2D cardiac CSE-MRI exam, and a breath-hold 3D liver CSE-MRI for determining liver iron concentration (LIC) using complex R2* mapping algorithm [29]. For R2* values below 400 s^−1^, we demonstrated that there is excellent reproducibility between these two methods for measuring liver R2*. For R2* values greater than 400 s^−1^, we demonstrated that R2* is underestimated by the 2D cardiac CSE-MRI.

The goal of this work is similar to a recent study Serai et al. that demonstrated good agreement between 2D cardiac and 2D liver CSE-MRI for R2* values from about 20 up to about 600 s^−1^ covering an equivalent R2* range as our study [22]. The work by Serai, however, used a magnitude-based R2* mapping method that does not correct for the presence of fat or noise. Thus, the reconstruction method used in our study, while related, is fundamentally different from their approach [22]. We also used a dedicated confounder-corrected complex 3D CSE-MRI method correcting the presence of fat and residual phase errors due to eddy currents as a reference standard rather than a 2D magnitude-based method [27, 28]. This complex reconstruction algorithm enables reliable R2* mapping in the liver but since it compensates for fat it is applicable to pancreas R2* mapping allowing reliable LIC determination in fatty pancreas [30, 31].

The Bland–Altman analysis in our study demonstrated good agreement between liver R2* measurements between a 2D cardiac ECG-gated and 3D breath-hold liver up to about 400 s^−1^ (Fig. 6). R2* values above 400 s^−1^ are underestimated using the 2D acquisition in comparison to the 3D acquisitions. Serai et al. could not demonstrate a comparable effect but in contrast they applied 2D sequences for both the dedicated liver and cardiac sequences [22]. Presumably, both 2D sequences underestimate the iron content for higher concentrations and the systematic error does not become obvious.

The important factor impacting the dynamic range of R2* determination in CSE-imaging is the choice of the sequence parameters, notably first echo time, but also echo spacing and number of echoes. Minimizing both the first and second TE is important to maximize the upper limit of the dynamic range of R2*, before the signal decay runs into the noise floor [29]. The combined choice of first TE and the following second echo defined by echo spacing, the first two echoes, is important to correctly fit R2* signal decay over the highest dynamic range possible.

We note that the first echo of our 2D acquisition was longer than the 3D method, related to the need to apply a slice selective 2D excitation pulse, which in general takes longer than in 3D. Additionally, the use of complex R2* reconstruction algorithm requires full echo acquisition to capture the phase information of the image. Therefore, shortening first TE through the use partial readout, which requires homodyne reconstruction that discards image phase [32], should be avoided. Ultimately, this limits the dynamic range of the 2D method to a narrower range of R2* values.

As Zhu et al. have recently shown in a phantom study, reliable measurement of R2* using 2D and 3D CSE-MRI depends on the signal-to-noise (SNR) ratio [33]. In their study, the relationship between R2* and iron content was highly linear for R2* values up to 1200 s^−1^, for both 2D and 3D CSE-MRI, so long as high SNR source images were used. However, when the SNR performance was similar to that expected with clinical 2D and 3D CSE-MRI acquisitions, the relationship between R2* and iron content only showed a linear relationship up to 400 s^−1^ and 500 s^−1^, for 2D and 3D acquisitions, respectively [33].

Altogether, the lower SNR performance and the longer first echo time of the 2D CSE-sequence leads to underestimation of R2* above 400 s^−1^ using the 2D cardiac CSE-MRI protocol. For R2* values below 400 s^−1^ this work demonstrates excellent reproducibility of R2* mapping to quantify liver iron overload. We note that an R2* of 400 s^−1^ at 1.5 T corresponds to severe iron overload, with an LIC of approximately 10.4 mg/g dry liver [22, 34].

The main limitation of our study is the moderate number of subjects in the two sub-studies over a broad range of R2* values, particularly for those subjects with R2* values above 400 s^−1^. Thus, a more detailed statistical analysis of this 7 subject group in comparison to the other two subgroups could not be performed. Only small differences between the defined subgroups with the exception of the slope in the linear regression analysis were observed. The linear regression analysis was not able show differences between the subpopulations since there are significantly fewer measurements above 400 s^−1^ than below 400 s^−1^.

A strength of our study is the combination of a retrospective and a prospective study which have demonstrated comparable results. The inclusion of patients as young as 2 months and as old as 71 years across the two sub-studies and the two R2* mapping methods have contributed substantially to the variability of the sequence parameters.

Reliable iron content quantification in the target organs is mandatory to initiate chelation therapy and for treatment monitoring, as iron deposition can lead to severe conditions like liver disease, endocrine disorders, cardiomyopathy, pancreatic insufficiency, and ultimately death [3]. Iron-chelating therapy should ideally be initiated before clinically significant iron accumulation occurs [3]. Despite the underestimation of R2* measurements on the 2D sequence for higher R2* values in comparison to the 3D sequence, the 2D exam may be sufficient for making appropriate clinical decisions, even when it is supplied as a secondary measure in a cardiac CSE-MRI exam [22]. The non-invasive nature of this method should facilitate the translation of this method to routine clinical use.

In summary, this study demonstrates that the quantification of liver iron with quantitative R2*-mapping is reproducible compared to 3D CSE-MRI R2* mapping with variable sequence parameters. R2* values above 400 s^−1^ may be underestimated using the 2D cardiac CSE-MRI protocol, likely related to the lower SNR performance and use of a longer first echo time of 2D acquisitions, both limiting its dynamic range. When these limitations are accounted for, acquiring and interpreting 2D cardiac R2* mapping can provide valuable clinical information about liver iron content. Given the higher dynamic range for R2* at high iron concentrations and breath-hold acquisition time, we recommend the use of a dedicated 3D liver-optimized CSE-acquisition for R2* mapping in the liver.
